# A smartphone-assisted brief online cognitive-behavioral intervention for pregnant women with depression: a study protocol of a randomized controlled trial

**DOI:** 10.1186/s13063-021-05179-8

**Published:** 2021-03-23

**Authors:** Pedro Fonseca Zuccolo, Mariana O. Xavier, Alicia Matijasevich, Guilherme Polanczyk, Daniel Fatori

**Affiliations:** 1 Department of Psychiatry, University of Sao Paulo Medical School, Sao Paulo, Brazil; 2 Department of Social Medicine, Post-Graduate Program in Epidemiology, Federal University of Pelotas, Pelotas, Brazil; 3 Department of Preventive Medicine, University of Sao Paulo Medical School, Sao Paulo, Brazil

**Keywords:** Maternal depression, Digital technology, Smartphone application, Cognitive behavioral therapy, Randomized controlled trial

## Abstract

**Background:**

Pregnancy is strongly associated with increased risk for depression. Approximately 25% of pregnant women develop depression. Treatment for depression during pregnancy has several complexities: the use of psychiatric medications during pregnancy might result in developmental problems in the child and must be used with caution. Psychosocial interventions are effective, but they require specialized professionals. Low- and middle-income countries (LMIC) such as Brazil do not have enough mental health professionals needed to meet this demand. In this context, smartphone-based interventions show immense potential. We developed Motherly, a smartphone application (app) designed to treat maternal depression. We aim to test the efficacy of Motherly in addition to brief cognitive-behavioral therapies (CBT) to treat maternal depression.

**Methods:**

We will conduct a 2-arm parallel-randomized controlled clinical trial in which 70 pregnant women aged between 16 and 40 years with depression will be randomized to intervention or active control. The intervention group will have access to Motherly, a smartphone app based on three concepts: psychoeducation, behavior monitoring, and gaming elements. Motherly is composed of a package of interventions composed of modules: mental health, sleep, nutrition, physical activity, social support, prenatal/postnatal support, and educational content. The main focus of Motherly is delivering behavioral activation (BA), a brief and structured psychological treatment. The app allows participants to schedule and engage in, and monitor activities according to a plan to avoid acting exclusively according to their mood. The active control group will have access to a simplified version of the app consisting of educational content about various aspects of pregnancy, maternal physical and mental health, and infant development (BA, activity scheduling, sleep hygiene, among other functionalities, will not be present in this version). Both groups will receive four sessions of brief CBT in 8 weeks. Participants will be evaluated by assessors blind to randomization and allocation status. Assessments will occur at baseline (T0), midpoint (T1, week 4–5), posttreatment (T2, week 8), and follow-up (T3, when the child is 2 months old). Maternal mental health (prenatal anxiety, psychological well-being, perceived stress, depression, depression severity, and sleep quality), quality of life, physical activity levels, and infant developmental milestones and social/emotional problems will be measured. Our primary outcome is the change in maternal prenatal depression from baseline to posttreatment (8 weeks).

**Discussion:**

The potential of digital technology to deliver mental health interventions has been increasingly recognized worldwide. There is a growing literature on interventions using smartphone applications to promote mental health, both with or without the intermediation of a mental health professional. Our study adds to the literature by testing whether an app providing an intervention package, including CBT, psychoeducation, nutrition, physical activity, and social support, can promote maternal and child health and well-being. In particular, we aim to treat depression, for which the use of digital technologies is still scarce. Smartphone applications designed to treat maternal depression are especially relevant because of the potential to circumvent barriers that prevent pregnant women from accessing mental health care.

**Trial registration:**

ClinicalTrials.gov NCT04495166. Prospectively registered on July 29, 2020.

## Background

Depression is currently one of the leading causes of years lived with disability worldwide [1]. Studies have shown that less than a third of people diagnosed with mental disorders such as depression receive any treatment [2, 3]. If left untreated, depression can lead to worsening of symptoms, the emergence of comorbidities, and loss of productivity [4, 5]. Pregnant women are at an increased risk of developing depression. Studies show that approximately 13% of pregnant women will have an episode of depression [6]. In low- and middle-income countries (LMIC), the prevalence of perinatal depression is estimated to be 25%, that is, higher than in high-income countries due to various socioeconomic and environmental factors known to be more prevalent in such countries (e.g., poverty, early life abuse, interpersonal violence) [7]. In LMICs, perinatal depression is a challenging public health problem because the number of health professionals is often not sufficient to meet the high demands for screening, diagnosis, and treatment. A study conducted in the UK estimated the economic cost of maternal depression to be around £8.1 billion per year, with 72% of the costs related to child outcomes [8]. No study investigated these costs in LMICs, but it can be hypothesized to be substantially higher due to the elevated prevalence of depression and less developed health systems in these countries.

The presence of risk factors can play a major role in the onset of maternal depression. Early life abuse, intimate partner violence, low educational attainment, low socioeconomic status, lack of social support, and history of mental health problems are known to increase the risk of developing depression [7]. The effects of maternal depression are not limited to individual impairment, distress, and loss of productivity, but have shown to be associated with long-lasting negative effects on the child. Children born of depressed mothers have an increased risk of low birth weight, stunting and delayed cognitive and motor development in the first years of life, behavioral problems at 3 years of age, poor school performance, and onset of mental disorders at 6 years of age, as well as depression during adolescence and adulthood [9–15]. A number of highly prevalent risk factors in LMICs, such as food insecurity, adverse living conditions, and lack of sanitation, may interact with these outcomes and increase the risk of worse psychopathological and developmental trajectories. This data shows that maternal depression should be treated as a public health priority.

The treatment of maternal depression during pregnancy has many complexities. Some studies suggest that psychiatric medication with well-documented efficacy and tolerability to treat depression may have teratogenic effects [16–18] and should be used with caution. Psychosocial treatments have been developed and tested, demonstrating efficacy in treating maternal depression using strategies such as psychotherapy and home visiting programs [19–22]. However, even though psychosocial interventions are efficacious and safe for the child, they demand specialized health professionals, particularly scarce in the public health system. LMICs are known to have two major issues that can prevent the implementation of such interventions: insufficient workforce of mental health professionals and lack of adequate financial investment in mental health programs [23, 24].

In addition, the current widespread model of screening and treatment delivery is based on the notion that patients must be aware of depressive symptoms to seek help or be referred by a health professional [25]. In the context of mental health, this model lacks scalability, reach, and affordability to deal with such a complex public health challenge. Innovative strategies must go over and beyond this model, also circumventing the lack of human and financial resources that are common in LMICs. A promising strategy with immense potential is to deliver mental health interventions via smartphones with or without the assistance of a mental health specialist. In the last years, the number of smartphone owners worldwide reached 2.5 billion, already surpassing this mark in 2020 [26]. Moreover, the global average price of smartphones is decreasing [27], quickly turning these devices into a low-cost portable computer with capabilities to deliver health interventions. Smartphones are already an integral part of everyday life, providing not only communication through voice calls and messaging, but endless possibilities, from calendars, organizers, and productivity enhancers to digital games, music, and video.

Studies have shown that mental health interventions delivered via smartphones are efficacious to treat a number of different conditions, such as depression [28, 29], anxiety [30], insomnia [31], and stress [32], among others. Researchers have been successfully adapting decades-old techniques commonly used in cognitive behavioral therapies (CBT) to the context of smartphone applications (apps). However, the potential of apps in preventing and treating mental health problems among pregnant women is yet to be fulfilled.

To our knowledge, few studies have tested the efficacy of an app to reduce depression symptoms [33]. Taiwanese researchers developed a social support intervention named Line. One-hundred and twenty-six pregnant women were enrolled at 36 weeks of gestation and randomly assigned to intervention or control. Women who used the Line app showed less perceived stress and postpartum depression symptoms when compared to the control group [34]. Another study conducted in Hong Kong tested an app focused on psychoeducation named iParent [35]. The app offered numerous articles on nutrition, childcare, and vaccines, among others, as well as videos dedicated to childbirth. Also, women in the intervention group were able to ask questions via app, which in turn were responded by obstetricians. Six-hundred and sixty pregnant women were randomized to intervention or control (treatment as usual). The authors reported a small effect size of the intervention on depression symptoms measured by the Edinburgh Postnatal Depression Scale (EPDS) at follow-up (postnatal period).

Despite the reduced number of studies using health apps for pregnant women, between Google’s Play Store and Apple’s App Store, hundreds of apps can be found using search terms related to pregnancy. However, the absolute majority was not tested using rigorous scientific methods. There is a growing debate among researchers and policymakers regarding the quality evaluation and certification of health apps. Currently, most apps are not meeting the needs of patients and clinicians, especially regarding safety, efficacy, and security [36, 37]. Health apps should be evaluated with the same rigor as other types of interventions, such as pharmacotherapy and psychotherapy. Given this gap in the literature, we developed Motherly, a smartphone app designed to treat maternal depression, as well as to promote maternal general mental health and infant development. We describe here the study protocol of a randomized controlled trial (RCT) designed to test the efficacy of an intervention delivered using this smartphone app in addition to brief online CBT to treat maternal depression.

### Objectives and outcomes

The efficacy of the Motherly app in conjunction with brief online CBT (intervention) will be evaluated in a RCT in comparison with an app designed to offer educational content about gestation, maternal health and mental health, and child development in addition to brief online CBT (active control).

The main objective of the study is to test the efficacy of a mental health intervention delivered via smartphone application in addition to brief online CBT to reduce depressive symptoms and to promote maternal and child health and well-being in pregnant women with depression. Our primary outcome is the change in maternal prenatal depression from baseline to posttreatment (8 weeks). We will also test the efficacy of the intervention on secondary outcomes assessed during gestation: change in maternal mental health (prenatal anxiety, psychological well-being, perceived stress, depression, depression severity, and sleep quality) from baseline to posttreatment, change in maternal prenatal quality of life from baseline to posttreatment, and change in maternal physical activity levels from baseline to posttreatment. Additionally, we will conduct a postnatal follow-up when the child is 2 months of age to test the impact of the intervention on the following secondary outcomes: change in maternal mental health (postnatal depression symptoms, depression severity, anxiety, psychological well-being, perceived stress, and sleep quality) from baseline to follow-up, change in maternal postnatal quality of life from baseline to follow-up, infant developmental milestones, and social/emotional problems at 2 months of age.

### Hypotheses

Participants receiving the Motherly app in addition to brief online CBT will show significantly greater reduction in symptoms of depression, as well as greater well-being, better overall mental health, and levels of physical activity compared with participants who receive only educational content in addition to brief online CBT. We also predict that children of mothers from the intervention group will show better development in the first 2 months of age in comparison with children from participants in the active control group.

## Methods

### Setting and design

We plan to conduct a 2-arm parallel-randomized controlled clinical trial (RCT). We aim to include 70 pregnant women between 16 and 40 years old. Since all interventions will be conducted online, participants will be recruited from any Brazilian state or municipality. Participants will be randomly assigned to either receive intervention via app consisting of behavioral activation (BA) and psychoeducation to promote changes in sleep, nutrition, and physical activity habits, as well as to engage in prenatal care, breastfeeding, and social support, and to stimulate child development, in addition to brief online CBT (*n* = 35); or to an active control group receiving an educational app with content about gestation, maternal health and mental health, and child development in addition to brief online CBT (*n* = 35). Duration of treatment will be 8 weeks, during which participants in both groups will be assessed at the beginning (baseline; T0), weeks 3–4 (midpoint; T1), and week 8 (endpoint; T2) in order to evaluate treatment effects. We will also conduct a follow-up postnatal assessment when the child is 2 months of age (T3).

### Inclusion and exclusion criteria

We will include pregnant women with the following characteristics: (a) aged between 16 and 40 years; (b) having a score of > 7 on the Edinburgh Postnatal Depression Scale (EPDS); (c) gestational age between 17 and 26 weeks; (d) being literate; (e) owning a functional smartphone with Android for personal use. Participants will be excluded if they meet one of the following conditions: (a) pregnancies classified as being at risk (e.g., hypertension, diabetes), fetal malformation, or congenital disease, (b) visual, auditory or intellectual disabilities, or chronic diseases associated with fetal development alterations, or (c) severe and/or chronic mental illness (e.g., schizophrenia, bipolar disorder).

### Procedures

#### Recruitment and randomization

We will use social media (Facebook, Instagram) advertisements, email, and WhatsApp messages to divulge our study and recruit participants. Women interested in participating in our study will be referred to a website page where they will be provided with written information on the study’s aims, procedures, and data collection. Afterwards, participants will be required to respond to an online survey containing questions related to eligibility criteria of the study. Eligible participants will be invited to a baseline assessment which will be conducted via internet or telephone. In the baseline assessment, after obtaining informed consent, the participant will be instructed to download the Motherly and register a profile with basic information. Randomization will occur automatically in real time using PHP 7 rand function at the end of registration process [38]. After the first login, the participant will already be using one of the two versions of the Motherly app (intervention or active control).

Participants cannot be blinded due to the nature of the intervention. Likewise, blinding of psychologists delivering brief online CBT will not be possible. However, all assessments will be conducted by assessors blind to randomization and allocation status.

#### Assessments and instruments

All assessments will be conducted via the internet (videotelephony) or telephone. Assessment instruments as well as time points at which they will be used are shown in Table 1. At baseline (T0), we will collect sociodemographic information (socioeconomic status, educational level, occupation, income) as well as frequency of use of smartphones, media, and apps. We will also conduct a comprehensive clinical assessment of maternal physical and mental health, perceived stress, personality traits, sleep quality, quality of life, physical activity frequency, and psychological well-being using the following instruments. We describe each instrument that will be used in the assessments below.

**Table 1:** Schedule of enrollment, interventions, and assessments

	Prenatal				Postnatal
	Enrolment	Baseline	Midpoint	Endpoint	Follow-up
	–	Week 0	Week 4–5	Week 8	2 months
	−T1	T0	T1	T2	T3
**Procedures**					
Recruitment, eligibility screening	X				
Randomization and allocation		X	X	X	
Both interventions		X	X	X	
**Assessments**					
Demographics		X			
Prenatal care		X	X	X	
Mental health treatment		X	X	X	
Use of smartphones, social media, and apps		X			
Edinburgh Postnatal Depression Scale (EPDS)	X	X	X	X	X
Generalized Anxiety Disorder 7 (GAD-7)		X	X	X	X
Drugs and Alcohol (modified ASSIST)		X			
Perceived Stress Scale (PSS)		X	X	X	X
Single-Item Measures of Personality (SIMP)		X			
Single-item sleep quality scale		X	X	X	X
12-item health survey (SF-12)		X		X	X
International physical activity questionnaire (IPAQ) Short Version		X		X	
Ryff’s Psychological Well-Being Scale		X		X	X
System Usability Scale (SUS)				X	
Mobile App Rating Scale: User version (uMARS)				X	
Survey of Wellbeing of Young Children (SWYC)					X
Postnatal care					X

The Edinburgh Postnatal Depression Scale (EPDS), a 10-item scale developed to assess pre- and postnatal depression. It is the most used scale in studies about depression during pregnancy and the postnatal period, largely used in observational studies and clinical trials aiming to measure presence and severity of depressive symptoms. It has been validated for use in Brazil and used in multiple studies throughout the years [12, 39–41].

The Generalized Anxiety Disorder Scale (GAD-7) is a 7-item scale that assesses anxiety disorders. Because it is brief and simple, this scale is suitable in primary care, especially when the objective is to track anxiety problems in the population. This scale also allows dimensional measuring of anxiety via a continuous score, thus serving as a severity measure [42]. It has been validated in Brazil showing good psychometric properties [43].

The Alcohol, Smoking and Substance Involvement Screening Test (ASSIST) is a scale developed by the World Health Organization to assess abuse or dependence of alcohol, tobacco, and other substances (e.g., cocaine, benzodiazepines, stimulants) widely used in Brazil [44]. This scale allows for both dimensional and categorical assessment of problems related to substance use. We will use only the first and second items of the ASSIST scale to assess lifetime and current substance use.

The Perceived Stress Scale (PSS) is one of the most used scales in the literature to assess general symptoms of stress. This scale has 10 items assessing the individual’s perception about stress throughout the previous months using a 5-point Likert scale. It was previously translated to Brazilian Portuguese and validated [45].

Single-Item Measures of Personality (SIMP), a scale which assesses personality based on the Big Five theory, which proposed five types of personality: openness to experience, conscientiousness, extraversion, neuroticism, and agreeableness. The SIMP utilizes five descriptions designed to provide a comprehensive evaluation of desirable and undesirable aspects of each personality dimension. It is a bipolar scale, that is, each dimension has extremes (positive and negative), each with its own description. The individual assigns how much he/she fits in each bipolar dimension representing the Big Five [46]. We translated the SIMP to Brazilian Portuguese for the purpose of using in this study.

Single-item sleep quality scale (SSQS), a 1-item scale ("How would you assess the quality of your sleep?") demonstrating high correlation with classical scales for assessing sleep problems in the literature, such as the Pittsburgh Sleep Quality Index (PSQI) and the Morning questionnaire-insomnia (MQI). Examinees can choose from 0 to 10, indicating his/her perception of the quality of sleep (from bad to excellent) [47]. Since the SSQS was not available in Brazilian Portuguese, we translated the scale to use in the present study.

The short version of the International Physical Activity Questionnaire (IPAQ) assesses daily physical activity. Twelve countries, including Brazil, participated in the validation of this scale. The IPAQ has been shown to be equivalent to other physical activity instruments and its data has been corroborated by objective measures via accelerometer, considered the gold standard in the literature [48]. Its robustness allowed for the assessment of physical inactivity in a study conducted in 17 countries with a sample of 130,000 participants [49].

The Ryff’s Psychological Well-Being Scale is an instrument developed by Carol Ryff based on the Artistotelian construct of eudaimonia and state of the art psychological theories of happiness and well-being [50–52]. This scale assesses six dimensions that compose the state of well-being with 36 items: Positive Relations With Others, Autonomy, Environmental Mastery, Personal Growth, Purpose in Life, and Self-Acceptance. The Ryff’s Psychological Well-Being Scale was validated in Brazil [53].

The 12-item health survey (SF-12) is a short version of the SF-36, a largely utilized scale to assess quality of life associated with health outcomes. It has a Likert scale format and assesses quality of life in two domains: physical and mental health. We used the second version of the SF-12, previously validated to the context of the Brazilian population [54].

At midpoint (T1), we will conduct a comprehensive clinical assessment of maternal physical health, and assess depression (EPDS), anxiety (GAD-7), perceived stress (PSS), and quality of sleep (SSQS), as well as prenatal and health services use.

At post-intervention (T2), 8 weeks after enrollment, we will conduct a clinical assessment with all instruments used in baseline, with the exception of the SIMP. In addition, we will administer the [55] and the Mobile App Rating Scale (MARS) [56] to assess the overall user experience of the Motherly app (intervention and active control).

At follow-up (T3), infant developmental milestones (motor, cognitive, communication/language) will be assessed when the child is 2 months old using the Survey of Wellbeing of Young Children (SWYC). Common pediatric symptoms will also be assessed using the Baby Pediatric Symptom Checklist (BPSC) section of the SWYC. The SWYC was validated in Brazil [57].

All assessments will be conducted by a team of psychologists with expertise in mental health and early childhood development with previous experiences in conducting assessment in clinical trials (NCT02807870, NCT04362098). After assessments at every time point, these professionals will be required to fill questions related to the quality of interview in terms of participant’s comprehension, collaboration, and quality of internet or telephone connection. Likewise, psychotherapists will fill questions related to the quality and fidelity of intervention after each session with the participant.

In addition to assessments conducted by assessors via the internet and/or telephone, we will measure depression, sleep problems, physical activity, and diet quality bi-weekly via app throughout the RCT. Assessment of symptoms of depression and anxiety will be conducted using the Patient Health Questionnaire for Depression and Anxiety (PHQ-4), a reduced version of the PHQ-9 [58] consisting of two questions and which has been validated in Brazil [40, 43, 59]. Sleep problems will be assessed using the Bergen Insomnia Scale [60], which was developed based on the Diagnostic and Statistical Manual of Mental Disorders (DSM-IV) [61]. The first four items assess the difficulty to initiate sleep, maintain sleep, awaken, and non-restoring sleep, and the last two assess functional impairment associated with quality of sleep. Physical activity will be evaluated daily using step counting via smartphone accelerometer, a device that measures the acceleration or force applied on the smartphone, allowing to determine the position of the smartphone on the tridimensional space. Raw data collected by the accelerometer are then converted into human steps using computational algorithms trained by means of statistical methods [62]. Thus, it is possible to assess in real time the level of physical activity of an individual in a passive and objective way. Step counting has been the most utilized method in recent studies on physical activity and sedentarism prevention [63, 64].

All instruments were already previously translated and validated in Brazil with the exception of the SIMP and SSQS. These two instruments were translated by a professional certified translator for the purposes of the current study. We plan to use data collected in our study to verify psychometric properties of these instruments, such as internal consistency and factor structure, among others.

#### Intervention: motherly app + brief online CBT

The intervention group will have access to Motherly 1.0, a mobile app designed to promote life habits that have been shown to improve physical and mental health in pregnant women. The Motherly 1.0 was developed by a team of psychologists, nutritionists, and app developers to translate treatment components into a mobile platform. The Motherly app was developed using the engine Unity to take advantage of enhanced graphical effects. The app consists of a package of specific and customized interventions defined by eight different modules: (1) Mental Health; (2) Sleep; (3) Nutrition; (4) Physical activity; (5) Social support; (6) Prenatal support; (7) Postnatal support, and (8) Library of pre- and postnatal content.

**Fig. 1:**
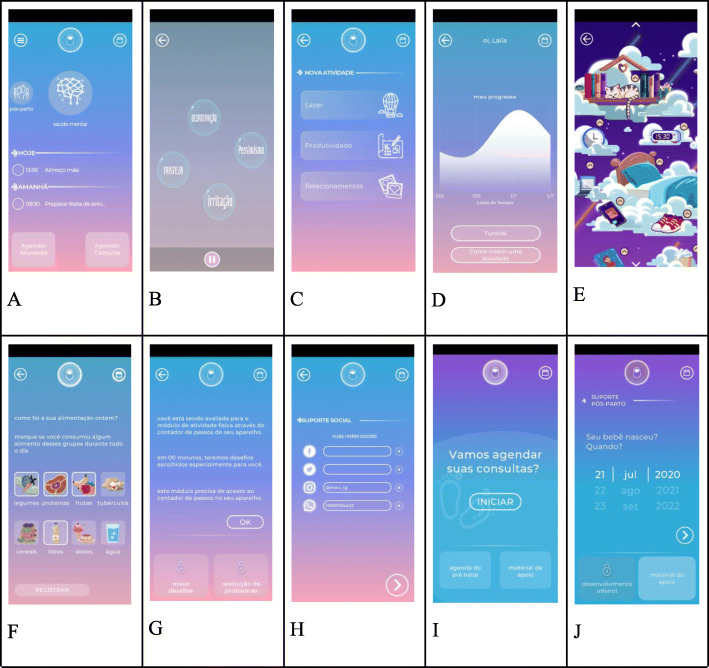
Screenshots of Motherly 1.0. **a** Main menu. **b** Behavioral Activation module (activities menu). **c** Behavioral Activation module (psychoeducation). **d** Behavioral monitoring. **e** Nutrition module. **f** Sleep module. **g** Physical activity. **h** Social Support. **i** Prenatal content. **j** Postnatal content

The aforementioned modules were integrated into a single interface (Fig. 1a) using three main concepts: psychoeducation, behavior monitoring, and gamification elements. Psychoeducation is delivered in four ways: (a) tutorials explaining the rationale for the intervention and showing how to use each module; (b) psychoeducational content related to health and pregnancy delivered as brief notifications and available in a library that can be read at the users’ discretion; (c) brief troubleshooting messages, which are suggestions of strategies to overcome difficulties with BA activities. Behavior monitoring is promoted using schedules, checklists, and notifications to help participants keep track of their health care visits, and schedule activities that have been associated with prevention and/or reduction of depressive symptoms. Gamification elements, defined as the use of game design elements in non-game contexts to engage users in problem-solving-behavior, are available to maintain and motivate healthy behaviors. Gamification elements are based on the psychology of motivation, behavior analysis, and game design theory [65, 66]. Specifically, the Motherly 1.0 app uses resources such as changes in the appearance of the background and icons so as to reflect participant’s mood assessment (described below) and ratings of activities, and graphical and easy-to-use questionnaires for obtaining information (mood, nutritional habits). These elements, along with psychoeducational messages and user’s responses, act as a reinforcement for app utilization.

In what follows, we provide descriptions of all modules as well as the rationale for developing them.

##### Mental health

This module is an adapted and automated version of BA, a brief and structured psychological treatment based on behavioral theories of depression [67, 68]. According to these theories, depression is associated with complex interactions between individual and environmental vulnerabilities, a lack of reinforcement for nondepressed behaviors, and increased escape/avoidance behaviors that maintain depressive behaviors [69]. BA assumes that depressive symptoms might be alleviated by engaging patients in behaviors that they will ultimately find productive or pleasurable, or that might improve their life situations providing greater rewards. Therefore, BA is action-oriented and focused on problem-solving, which requires patients to test new ways of behaving in daily situations and in different areas of their lives. Specifically, BA focuses on promoting behavioral change to diminish depressive symptoms by (a) engaging patients in positively reinforced behavior (which in many cases consists in involving in activities resulting in experiences of mastery and/or pleasure), (b) decreasing avoidance/escape behaviors that maintain depression, and (c) improving problem-solving skills in order to increase access to reward and prevent depressive symptoms [69]. Decades of clinical research supports the efficacy of BA to treat depression in general populations [70–74] with some studies showing comparable efficacy relative to antidepressant medication [75–77] or more complex forms of psychotherapy [78]. In two studies, BA has been shown to be as effective as antidepressant medication in treating depressive symptoms but with superior retention [79] and enduring effects over a 2-year follow-up [77]. Given its simplicity, BA has been successfully delivered by nonspecialists [80, 81], as well as via internet, computer, or smartphone [29, 82, 83]. In a recent RCT, BA was effective in diminishing depressive and symptoms, as well as perceived stress in pregnant women [75].

Based on two manualized versions of BA [84, 85], the mental health module was designed to assist users to schedule and engage in, and monitor activities according to a plan to avoid acting exclusively according to their mood. This module consists of five basic components: (1) psychoeducation, (2) generation of activities; (3) behavior monitoring and mood assessments; (4) problem-solving; (5) reinforcement for app utilization.

1. Psychoeducation: Users have access to an animated video tutorial presenting the BA rationale, specifically, explaining the connections between thoughts, actions, and feelings, and the ways in which they interact (Fig. 1b). Users are also shown how to use this module by means of a written tutorial. Brief psychoeducational messages are also used to provide users with information on inherent difficulties to engage in activities and common strategies to overcome them (problem-solving suggestions).

(2) Activity scheduling: Users can schedule activities in three main life areas: recreation, relationship, and productivity (i.e., activities related to the daily routine, career, education, and health, including activities related to other modules within Motherly, as described ahead) (Fig. 1c). The app has a list of suggestions for activities that can be edited to fit the users’ needs. When generating (or editing) activities, users have an option to insert a brief sentence describing a motivation to engage in that particular activity (“This activity is important to me because …” ). Like the activities, the app provides an initial editable list of suggestions for motivations. The lists of suggestions for activities and motivations were developed based on the Brief Behavioral Activation Treatment for Depression: Revised Treatment Manual (BATD-R) [85] manual as well as on the clinical experience from one of the authors (PFZ) with depressive patients and aimed at increasing the likelihood that users choose activities that are actually reinforcing and objective (e.g., “buying clothes for my baby,” “message my friend”), avoiding general objectives that are too hard or cannot be attained in the short term (e.g., “feeling better about myself,” “be a great mother”). Therefore, activity lists contain activities that are time-limited, preferably observable by others. Motivations are made available so that users choose activities that are closely linked with values, ideals, or qualities they think are important. Considering the difficulty that some patients have to complete planned activities, the Motherly also has an option to insert the contact and/or name of a person that could support in completing the activity.

(3) Activity engagement and problem-solving: Activities can be organized in a calendar. When users register activities as completed they are prompted to evaluate their sense of mastery and the difficulty to complete the activities. When participants register activities as not completed, the app provides brief troubleshooting psychoeducational messages. Specifically, participants can choose a list of reasons for not completing the activity, which will be followed by a brief and specific suggestion of strategy to overcome that difficulty. The lists of suggestions for strategies were created based on common recommendations that clinicians give patients when they face difficulties in pursuing therapeutic goals in a BA context.

(4) Mood assessments: Users are prompted to report their mood bi-weekly by means of completing an abbreviated version of PHQ. Users also have the option to complete abbreviated mood assessment whenever they want.

(5) Behavior monitoring: Responses to mood assessments are available in a graph to allow users to monitor their progression. The *x*-axis is time and the *y*-axis the scores (Fig. 1d). Modifications in the background color app’s icons are also used to help users to keep track of their progress.

(6) Reinforcement for module utilization: Gamification elements such as changes in the appearance of the app so as to reflect participant’s mood assessment, as well as psychoeducational messages, are sent to motivate app utilization. Color saturation is increased (less symptoms) or decreased (more symptoms) depending on self-reports.

##### Sleep

This module was designed based on two techniques that are frequently part of CBT protocols for insomnia: sleep hygiene and relaxation [86]. Sleep hygiene (SH) is a psychoeducation-based intervention in which patients are provided information about lifestyle (diet, exercise, substance use), environmental (temperature, noise, light), and behavioral (e.g., the time spent lying in bed) factors that might interfere or promote better sleep [86, 87]. SH has been shown to improve sleep quality in adults with insomnia [88] and has already been shown to be effective when delivered via apps [31]. Relaxation techniques consist in procedures that decrease somatic and cognitive arousal. These procedures are especially useful for onset insomnia, since some level of arousal is present in the majority of these cases [89]. Relaxation techniques described in Motherly were based on progressive muscular relaxation (PMR) and deep breathing, which have been shown to improve sleep in multisite clinical trials with pregnant women and young mothers [90, 91]. PMR involves alternately tensing and relaxing different muscle groups while patients are instructed to focus and compare feelings of relaxation and tension [92, 93]. Deep breathing aims to direct the patient’s attention to breathing rhythm in order to diminish arousal [86]. Motherly users have access to sleep hygiene and relaxation procedures that are presented in the form of short audio explanations along with brief texts and visual stimuli to guide users throughout each technique (Fig. 1e).

##### Nutrition (Fig. 1f)

Healthy nutrition during pregnancy is critical to maternal health and fetal development [94] and can also influence the onset of long-term diseases [95]. In addition, having good eating habits makes it easier for pregnant women to gain weight within the recommended limits [96], which is associated with better maternal and infant outcomes [97]. Diet can also influence mental health [98, 99] and evidence suggests that increasing consumption of fruits, vegetables, legumes, whole grain cereals, healthy fats, such as nuts and seeds, and lean proteins, including fish is associated with a reduced risk for depression [100]. Therefore, the aims of the nutrition module are to improve nutritional habits and to promote healthy gestational weight gain. The intervention includes self-monitoring, feedback feature, and push notifications. Diet and weight gain are reported by the user at baseline and every 30 days until delivery, which will be followed by tailored feedback in the form of text messages.

Information about dietary habits will be derived from a short food frequency questionnaire, consisting of 14 items developed for the study based on the current food guide for the Brazilian population [101] and the guide for pregnant women from the Brazilian Ministry of Health [102]. There are 13 food groups: total grain/cereal/roots and tubers, whole grain/cereal, vegetables, dairy products, artificial juices/soft drinks, fast-food/processed foods/salty snacks, fruits; beans, oils, nuts/seeds, fish, meat/eggs, sugar/sweets, and one item about alcoholic beverages. The questions about the frequency of consumption of these food groups will be asked regarding the week prior to assessment, and the answer options are “every day,” “5–6 times per week,” “2–4 times per week,” “1 time per week,” and “never.”

The feedback feature and push notifications provided are also consistent with the current food guide for the Brazilian population [101] and the guide for pregnant women from the Brazilian Ministry of Health [102]. According to these guidelines, the basis of the diet should come from fresh or minimally processed foods avoiding ultra-processed foods. Some food groups need to be consumed daily for a healthy diet (i.e., fruits, vegetables and legumes, dairy products, among others), and other food groups should be avoided (i.e., artificial juices/soft drinks, fast-food/processed foods/salty snacks, among others). Based on the aforementioned recommendations and on the answers provided, they will receive a score for each component after answering the questionnaire: (10) maximum score (reached the recommendation), (5) average score, (0) minimum score (far from reaching the recommendation). The maximum score is 140 representing 100% of adequacy. A score above 80% will be considered “good quality diet,” between 50 and 79% considered “need of some improvements” and below 50%, “need of deep improvements.” In addition to the final score, participants will also receive tailored feedback in text format for each component that did not reach the recommendation. Participants can also self-monitor food intake by answering a 24-h food recall based on food groups whenever they wish.

Regarding gestational weight gain, participants will be asked about their pre-gestational weight and height at baseline. Pre-pregnancy body mass index (BMI) will be calculated and the recommended weight gain range will be individually provided according to the Institute of Medicine classification [103]. Weight self-monitoring consists of reporting current weight, and weight gain is presented graphically. We will also analyze gestational weight gain within the Institute of Medicine-recommended levels by using the suggested weekly gains in body weight during the second and third trimesters of pregnancy [103].

##### Physical activity (Fig. 1g)

Regular physical activity promotes several health benefits [104]. High levels of physical activity (including both recreational and non-recreational) were associated with a lower risk of mortality and cardiovascular disease events in individuals from low-income, middle-income, and high-income countries [49]. During pregnancy, an exercise program has been shown to reduce the prevalence of depression in late pregnancy and postpartum [105]. However, a recent study including 358 surveys across 168 countries (1.9 million participants) identified a global age-standardized prevalence of insufficient physical activity of 27.5% [106].

According to the American College of Obstetrician and Gynecologists (ACOG), pregnancy is an ideal time for keeping or adopting a healthy lifestyle and women with uncomplicated pregnancies are encouraged to take part in aerobic and strength-conditioning exercises before, during and after pregnancy [107]. Besides promoting benefits related to physical health outcomes of mother and child, such as reduced risk of excessive gestational weight gain, lower likelihood of gestational diabetes mellitus, and lower likelihood of delivering a large-for-gestational-age infant [108], regular physical activity during pregnancy is also related to enhancing psychological well-being and may reduce the prevalence of depression in late pregnancy and postpartum [105].

The current recommendation for pregnant women is at least 20–30 min per day of moderate-intensity exercise every day or most days of the week (the same recommendation of at least 150 min per week for adults), and the exercise program should be adjusted as medically indicated [107]. Physical activity can be measured by objective monitoring using a pedometer or accelerometer. Pedometer use has been associated with increased physical activity in adults [109]. Some guidelines specifically recommend taking at least 10,000 steps per day as a sufficient physical activity for adults [64].

This intervention aims to encourage physical activity by monitoring steps via accelerometer and, subsequently, propose an increase in the average number of steps by walking. First, pregnant women are informed about the recommendations and benefits of regular physical activity during pregnancy. Then, they are invited to participate in the intervention. To avoid potential harm, exclusion criteria for the module are the following: (1) multiple pregnancy; (2) those with pre-gestational BMI < 12 kg/m^2^ or > 40 kg/m^2^; (3) known medical or obstetric complications which restrict physical activity.

The smartphone accelerometer will be used to assess the number of daily steps. After completing the first week, users will receive feedback about their level of physical activity according to the average number of steps for the first 7 days: sedentary (< 5000 daily steps), low active (5000 to 7499 daily steps), somewhat active (7500 to 9999 daily steps), and active (≥ 10,000 daily steps) [64]. Women will be encouraged to increase their steps by 10% each week until reaching 10,000 steps/day. Women who achieve the 10,000 steps will be encouraged to continue their activity level and no further increases will be proposed.

Pregnant women will be able to self-monitor their daily number of steps. At the end of each week, the weekly average of daily steps will be presented. A tailored feedback will be provided according to the user’s prescheduled weekly step goal, and a new goal will be established for the following week. The evolution will be graphically shown. If the pregnant woman is unable to meet her goal for at least three consecutive weeks, support will be provided considering the possible reasons that may have led to this situation and some ideas to circumvent the problems will be provided. For example, if the participant select the option “I don’t know how to increase my number of steps,” an answer will be provided: “How about starting to change small details in your daily life, such as, whenever possible, choosing stairs instead of elevator, going to nearby places on foot, extending the walk with your pet, enjoying sunny days to make pleasant walks on the street, among others.”

##### Social support (Fig. 1h)

Social support is known to be associated with morbidity and mortality [110]. During pregnancy, lack of social support is an important risk factor for maternal depression and low birth weight [111]. Interventions focused on increasing social support are known to have an effect on decreasing symptoms of depression [112]. Therefore, users will be able to make available their social media contacts in a list displayed in the app. This list can be used to contact other users.

##### Prenatal support (Fig. 1i)

In line with evidence showing that an adequate prenatal care can reduce child mortality and prevent several neonatal problems [113], Motherly provides a calendar to assist users in planning checkups from their doctors and prenatal exams according to current international guidelines [114]. Users receive notification to update their calendars, as well as register whether any health conditions have been diagnosed, thereby increasing the likelihood of receiving adequate prenatal care.

##### Postnatal support (Fig. 1j)

After child birth, users receive notifications describing the benefits of breastfeeding to child development and are prompted to say whether they are able to breastfeed their babies. They can register whether they are having difficulties breastfeeding, in which case the app provides a list of suggestions for solving potential issues.

##### Library of pre- and postnatal content

The Motherly app has a comprehensive collection of educational content. These brief articles have content related to depression and anxiety, nutrition, fetal development, breastfeeding, sleep, physical activity, among others, and can be consulted by users at their discretion.

Throughout the 8 weeks of treatment, participants in the intervention group will undergo brief online CBT with a focus on BA in four sessions. More specifically, psychotherapists will help participants to use Motherly’s functionalities that were developed to implement BA, that is, schedules, activity planning, hierarchization of activities according to their level of difficulty, selection of motivations for each activity, and use of problem-solving suggestions. In addition, they will help navigate the app and use other modules. Throughout the four sessions, psychotherapists will monitor participants’ adherence, answering questions about the strategies and providing support for solving problems or circumventing barriers to completing scheduled activities. CBT techniques such as cognitive restructuring, relaxation techniques, sleep hygiene, stress and anxiety management, among other evidence-based techniques might be used if appropriate to treat depression and possible comorbidities. The general structure of the sessions will be based on a guide developed by the authors (DF and PFZ) to help psychotherapists select CBT interventions that are adequate for each participant.

#### Active control: educational app + brief online CBT

Participants allocated to the active control group will have access to a simplified version of the app consisting of educational content about various aspects of pregnancy, maternal physical and mental health, and child development. Active intervention functionalities, such as behavioral activation, activity scheduling, and sleep hygiene, among others, will not be present in this simplified version. As with the intervention group, participants from the active control group will receive app notifications to answer questions related to the outcomes of this study. The active control group will also receive four sessions of brief online CBT throughout the 8 weeks, with a focus on BA. They will be guided by psychotherapists to plan, schedule, and engage in positively reinforcing activities, and will be aided to develop problem-solving strategies for circumventing barriers to completing scheduled activities. However, implementation of these techniques will be conducted without the aid of the Motherly app (as these functionalities are not present in the active control app). Throughout the four sessions, psychotherapists will monitor participants’ adherence, answering questions about the strategies and providing support for solving problems or CBT techniques, such as cognitive restructuring, relaxation techniques, sleep hygiene, stress and anxiety management, among other evidence-based techniques might be used if appropriate to the case. The structure of the sessions will also follow the guide mentioned previously.

#### Sample size

We calculated the sample size based on an expected effect size of 0.65 on depressive symptoms, which is based on a previous meta-analysis [115] (difference in means between two independent groups) considering a probability of type I error of 5%, statistical power of 80%, a two-tailed test, and a dropout rate of 15%.

#### Statistical analyses

First, continuous variables will be described using central tendency measures and categorical variables will be described using frequencies and cross tabulations. To analyze the potential impact of the intervention on depression symptoms, we will use an intention-to-treat (ITT) approach. To perform ITT analyses, we will use multiple imputation by chained equations to include every participant randomized. The effects of intervention on maternal depression (primary outcome) and secondary outcomes will be tested using generalized linear models. Estimated marginal means will be extracted from these models to describe primary and secondary outcomes of the study for both groups. Marginal means will be used to plot data. Additionally, we will investigate the potential role of moderators (baseline characteristics, such as socioeconomic aspects and personality traits) of the treatment effect on outcomes using a regression-based approach [116]. Standardized effect sizes will be calculated from the difference between group means using criteria described by Cohen [117]. Tests will be considered significant at *p* < 0.05 and 95% confidence intervals will be reported for all parameters. All analyses will be conducted using STATA 16 and R.

#### Data safety

All data from the assessments as well as clinical records from CBT sessions will be entered electronically via Research Electronic Data Capture (REDCap) [118], thereby ensuring safety of the data and protections of participants’ information.

#### Harms

Given that the intervention in this study is non-invasive, the risk for participants can be considered marginal. Participants will be informed that questions in the assessment protocol might cause some level of subjective discomfort, but that they are not obliged to answer them and will still continue in the study and receive treatment even if they choose not to answer them. Irrespective of the group to which participants will be allocated, they will benefit from receiving brief online CBT by a mental health expert, which can potentially alleviate symptoms of depression or anxiety.

#### Staff

All assessments will be conducted by a team of psychologists with expertise in mental health and early childhood development with experience in clinical trials. Assessors will receive training sessions with a focus on presenting study details, as well as in-depth explanations of all instruments used. Brief online CBT will be delivered by female psychologists with a certified specialization in CBT and previous experience in treating depression. In order to ensure fidelity and quality of treatment, psychotherapists will receive weekly supervision by a senior doctorate level clinical psychologist (PFZ) with clinical background in CBT and BA.

#### Trial management

This trial is monitored by three committees: a Trial Coordinating Centre, a Trial Steering Committee, and the Ethics Committee of the University of Sao Paulo Medical School. The Trial management committee is composed of the principal investigators, trial managers, and research team leads, who meet weekly to coordinate and monitor the progress of the trial, and to ensure that the protocol is adhered to. This committee is also responsible for monitoring and reporting to other committees any adverse event (and taking action to ensure safeguard of participants), as well as ensuring quality of the trial.

The Trial Steering Committee is composed of senior researchers from our group (GVP, AM, and DF) and meet once a month to oversee the trial, ensure that it is conducted according to the protocol, and monitor its progress regarding recruitment and retention of participants, the appearance of adverse events and actions needed to ensure safeguard of participants, and adherence to the timeline of the study. This committee is also responsible for making decisions about the continuation, modification, or termination of the study. If this committee decides to make modifications in the trial, these will only be carried out with approval from the Ethics Committee, in which case sponsors will be notified and the protocol will be updated in the clinical trial registry. The Trial Steering Committee also meets with sponsors every six months to report on the status of trial.

The Ethics Committee of the University of Sao Paulo Medical School is responsible for reviewing all procedures involved in this trial, assessing its relative risks, and determining the guidelines as to what action should be taken to ensure safeguard of participants. This board also reviewed and approved study protocols and informed consent prior to data collection. Since this intervention is considered low risk, no periodic meetings were considered. However, other meetings might be scheduled if any adverse event occurs, or in the event that the trial steering committee decides to make amendments to the trial protocol.

Given that this intervention was rated by the Ethics Committee as low-risk, no Data Monitoring Committee was considered.

#### Ethical issues and dissemination

Throughout the study, both intervention and active control groups will be assessed for symptoms of depression, anxiety, sleep, and dietary patterns via app. These data will be available to researchers in real time for monitoring purposes if participants need to be referred to specialized care due to severe symptoms of depression or suicidal ideation. In these cases, participants will be contacted by internet or telephone.

During treatment, participants will be referred to specialized care in case depression symptoms worsen to serious, with suicidal ideation, functional impairment, and appearance of psychotic symptoms, among others. Participants presenting mental health issues associated with the COVID-19 pandemic and/or social distancing will be treated with psychological first care techniques adequate to stress-related symptomatology due to adverse conditions [119]. At the end of 8 weeks, participants who do not show remission of symptoms of depression will be referred to specialized mental health services.

#### Trial registration and status

This is a protocol version 1.0, registered on 29th July 2020. Recruitment began August 2020 and ended October 2020. Primary outcome assessment was completed December 2020. Follow-up assessments have begun January 2021 and are anticipated to end by May 2021. Detailed information on the trial register is presented in Table 2.

**Table 2:** World Health Organization Trial Registration Data Set

Data category	Information
Primary registry and trial identifying number	ClinicalTrials.gov NCT04495166
Date of registration in primary registry	29/07/2020
Secondary identifying numbers	SB-POC-1810-20573
Source(s) of monetary or material support	Grand Challenges Canada Fundação Maria Cecilia Souto Vidigal
Primary sponsor	University of Sao Paulo
Secondary sponsor(s)	Grand Challenges Canada Fundação Maria Cecilia Souto Vidigal
Contact for public queries	Daniel Fatori, PhD (daniel.fatori@gmail.com)
Contact for scientific queries	Daniel Fatori, PhD, University of Sao Paulo Medical School
Public title	A Smartphone-Assisted Brief Behavioral Intervention for Pregnant Women With Depression
Scientific title	A Smartphone-Assisted Brief Behavioral Intervention for Pregnant Women With Depression
Countries of recruitment	Brazil
Health condition(s) or problem(s) studied	Perinatal depression
Intervention(s)	Treatment: Motherly app + brief psychotherapy Active control: Educational app + brief psychotherapy
Key inclusion and exclusion criteria	Ages eligible for study: 16 to 40 years Sexes eligible for study: female Gender based: no Accepts healthy volunteers: no Inclusion criteria: women aged between 16 and 40 years; having a score of > 7 on the Edinburgh Postnatal Depression Scale (EPDS); gestational age between 17 and 26 weeks; being literate; owning a functional smartphone with Android for personal use. Exclusion criteria: pregnancies classified as being at risk, fetal malformation, or congenital disease; visual, auditory or intellectual disabilities, or chronic diseases associated with fetal development alterations; severe and/or chronic mental disorder (e.g., schizophrenia, bipolar disorder).
Study type	Interventional Allocation: randomized Intervention model: parallel assignment Masking: Double (Investigator, Outcomes Assessor) Primary purpose: Treatment Phase III
Date of first enrolment	August 2020
Target sample size	70
Recruitment status	Recruitment ended
Primary outcome(s)	Change in maternal prenatal depression from baseline to posttreatment (8 weeks)
Key secondary outcome(s)	Change in maternal prenatal anxiety from baseline to posttreatment Change in maternal prenatal quality of life from baseline to posttreatment Change in maternal prenatal psychological well-being from baseline to posttreatment Change in maternal physical activity levels from baseline to posttreatment Change in maternal prenatal perceived stress from baseline to posttreatment Change in maternal prenatal depression severity from baseline to posttreatment Change in maternal prenatal sleep quality from baseline to posttreatment Infant developmental milestones at 2 months of age

## Discussion

The potential of digital technology to deliver mental health interventions has been increasingly recognized worldwide [120]. There is a growing literature on interventions using smartphone applications to promote mental health, both with [121, 122] or without the intermediation of a mental health professional [31]. Our study adds to the literature by testing whether an app providing an intervention package, including CBT, psychoeducation, nutrition, physical activity, and social support, can promote maternal and child health and well-being. In particular, we aim to treat depression, a condition which is associated with negative outcomes in both the mother and the offspring [123–125] and for which the use of digital technologies is still scarce. Smartphone applications designed to treat maternal depression are especially relevant in LMICs, such as Brazil, as they have the potential to circumvent barriers that prevent pregnant women to access mental health care, namely, the lack of specialized professionals and economic resources [120].

Despite the evident potential of our study, some challenges and limitations need to be acknowledged. First, behavioral activation, one the main components of this intervention, is a treatment which includes two aspects that are challenging to translate into the scope of an app: (1) the customization of goals and activities in order to meet the specific needs of individual patients, since what is a positive reinforcer for behavior usually vary from one individual to the other, and (2) the need to help patients develop problem-solving strategies to overcome difficulties in achieving these goals [69, 84, 85]. To face these challenges, the app was designed to allow for the customization of the activities (so as to meet individual needs), as well as the insertion of brief sentences describing the motivation for involving in a given activity. The aim of this functionality is to increase the chances of users choosing activities that are actually reinforcing and minimally objective, avoiding general objectives that cannot be attained. A list of brief troubleshooting psychoeducational messages was also developed based on common recommendations that clinicians give patients when they face difficulties in pursuing therapeutic goals within BA settings. This list can be accessed by patients when they register activities as not completed.

Finally, we chose to use brief CBT in addition to the app so as to ensure that these functionalities are properly used by participants. Aside from increasing treatment quality, psychotherapist monitoring of participants’ app usage will allow us to identify which of these functionalities are actually helpful and provide insights as to how we could improve them to be self-sufficient. Testing the Motherly app in addition to CBT was also based on an ethical decision, since offering an app without previous evidence of efficacy to depressed women without assistance could potentially do harm. Data derived from the present study will allow us to develop and deliver a more complete and definitive version of Motherly designed to be used by pregnant women without assistance of mental health professionals, potentially being an intervention to treat mild and moderate cases of depression. This new version will be key to reaching scalability of a low-cost intervention.

Due to the fact that the RCT will be conducted during the coronavirus pandemic in Brazil, all contact with participants, including assessments and CBT sessions, will be exclusively online and/or by phone. Given the heterogeneity in infrastructure between regions within the country, it is possible that assessors will face connection issues. In these cases, some CBT sessions and assessments might have to be conducted by phone, which might be challenging to establish rapport or more difficult to conduct assessments without visual cues. In the case of CBT, staff will be trained to reinforce therapeutic alliances in the first session to prevent dropout rates associated with poor internet connection and adherence issues associated with a lack of facial contact with the therapist.

One last challenge is related to adherence to treatment. A recent meta-analysis found that the mean dropout rate in RCTs testing smartphone apps targeting depressive symptoms is 26.8% and can increase up to 47.8% when accounting for publication bias [126]. These attrition rates are higher than the ones found for individual psychotherapy for depression (19.9%) [127] and might represent a threat to the assessments of the efficacy of app-based interventions. However, this same meta-analysis found that integrating a human component, for example, when the app involved human feedback, and that integrating mood monitoring in the platform diminish these dropout rates considerably (from 47.8% to approximately 18% and 11% respectively) [126]. Both strategies have been included in our study protocol, as participants will have the aid of a mental health professional, and bi-weekly mood assessments. Moreover, to increase the likelihood that participants will keep using the app, we have introduced gamification elements, as well as an easy-to-use interface, and conciseness of texts to fit social media style. We expect that these strategies will increase adherence, allowing an objective analysis of minimum dose necessary for treatment response. We also expect to analyze which of these gamification elements are the most engaging in order to improve future versions of the app.

## Data Availability

Any data required to support the protocol can be supplied on request. The datasets analyzed during the current study are available from the corresponding author on reasonable request.

## Abbreviations

ACOG: American College of Obstetricians and Gynecologists
ASSIST: Alcohol, Smoking and Substance Involvement Screening Test
BA: Behavioral activation
BATD-R: Brief Behavioral Activation Treatment for Depression
BMI: Body mass index
BPSC: Baby Pediatric Symptom Checklist
CBT: Cognitive behavioral therapies
DSM-IV: Diagnostic and Statistical Manual of Mental Disorders
EPDS: Edinburgh Postnatal Depression Scale
GAD-7: Generalized Anxiety Disorder Scale
IPAQ: Physical Activity Questionnaire
ITT: Intention-to-treat
LMIC: Low- and middle-income countries
MARS: Mobile App Rating Scale
MQI: Morning questionnaire-insomnia
PHQ-4: Patient Health Questionnaire for Depression and Anxiety
PMR: Progressive Muscular Relaxation
PSQI: Pittsburgh Sleep Quality Index
PSS: Perceived Stress Scale
RCT: Randomized Controlled Trial
SIMP: Single-Item Measures of Personality
SF-12: 12-item health survey
SSQS: Single-item sleep quality scale
SUS: System Usability Scale
SWYC: Survey of Wellbeing of Young Children
WHO: World Health Organization

## References

[1] James SL, Abate D, Abate KH, Abay SM, Abbafati C, Abbasi N, Abbastabar H, Abd-Allah F, Abdela J, Abdelalim A, Abdollahpour I, Abdulkader RS, Abebe Z, Abera SF, Abil OZ, Abraha HN, Abu-Raddad LJ, Abu-Rmeileh NME, Accrombessi MMK, Acharya D, Acharya P, Ackerman IN, Adamu AA, Adebayo OM, Adekanmbi V, Adetokunboh OO, Adib MG, Adsuar JC, Afanvi KA, Afarideh M, Afshin A, Agarwal G, Agesa KM, Aggarwal R, Aghayan SA, Agrawal S, Ahmadi A, Ahmadi M, Ahmadieh H, Ahmed MB, Aichour AN, Aichour I, Aichour MTE, Akinyemiju T, Akseer N, al-Aly Z, al-Eyadhy A, al-Mekhlafi HM, al-Raddadi RM, Alahdab F, Alam K, Alam T, Alashi A, Alavian SM, Alene KA, Alijanzadeh M, Alizadeh-Navaei R, Aljunid SM, Alkerwi A', Alla F, Allebeck P, Alouani MML, Altirkawi K, Alvis-Guzman N, Amare AT, Aminde LN, Ammar W, Amoako YA, Anber NH, Andrei CL, Androudi S, Animut MD, Anjomshoa M, Ansha MG, Antonio CAT, Anwari P, Arabloo J, Arauz A, Aremu O, Ariani F, Armoon B, Ärnlöv J, Arora A, Artaman A, Aryal KK, Asayesh H, Asghar RJ, Ataro Z, Atre SR, Ausloos M, Avila-Burgos L, Avokpaho EFGA, Awasthi A, Ayala Quintanilla BP, Ayer R, Azzopardi PS, Babazadeh A, Badali H, Badawi A, Bali AG, Ballesteros KE, Ballew SH, Banach M, Banoub JAM, Banstola A, Barac A, Barboza MA, Barker-Collo SL, Bärnighausen TW, Barrero LH, Baune BT, Bazargan-Hejazi S, Bedi N, Beghi E, Behzadifar M, Behzadifar M, Béjot Y, Belachew AB, Belay YA, Bell ML, Bello AK, Bensenor IM, Bernabe E, Bernstein RS, Beuran M, Beyranvand T, Bhala N, Bhattarai S, Bhaumik S, Bhutta ZA, Biadgo B, Bijani A, Bikbov B, Bilano V, Bililign N, Bin Sayeed MS, Bisanzio D, Blacker BF, Blyth FM, Bou-Orm IR, Boufous S, Bourne R, Brady OJ, Brainin M, Brant LC, Brazinova A, Breitborde NJK, Brenner H, Briant PS, Briggs AM, Briko AN, Britton G, Brugha T, Buchbinder R, Busse R, Butt ZA, Cahuana-Hurtado L, Cano J, Cárdenas R, Carrero JJ, Carter A, Carvalho F, Castañeda-Orjuela CA, Castillo Rivas J, Castro F, Catalá-López F, Cercy KM, Cerin E, Chaiah Y, Chang AR, Chang HY, Chang JC, Charlson FJ, Chattopadhyay A, Chattu VK, Chaturvedi P, Chiang PPC, Chin KL, Chitheer A, Choi JYJ, Chowdhury R, Christensen H, Christopher DJ, Cicuttini FM, Ciobanu LG, Cirillo M, Claro RM, Collado-Mateo D, Cooper C, Coresh J, Cortesi PA, Cortinovis M, Costa M, Cousin E, Criqui MH, Cromwell EA, Cross M, Crump JA, Dadi AF, Dandona L, Dandona R, Dargan PI, Daryani A, Das Gupta R, Das Neves J, Dasa TT, Davey G, Davis AC, Davitoiu DV, de Courten B, de la Hoz FP, de Leo D, de Neve JW, Degefa MG, Degenhardt L, Deiparine S, Dellavalle RP, Demoz GT, Deribe K, Dervenis N, Des Jarlais DC, Dessie GA, Dey S, Dharmaratne SD, Dinberu MT, Dirac MA, Djalalinia S, Doan L, Dokova K, Doku DT, Dorsey ER, Doyle KE, Driscoll TR, Dubey M, Dubljanin E, Duken EE, Duncan BB, Duraes AR, Ebrahimi H, Ebrahimpour S, Echko MM, Edvardsson D, Effiong A, Ehrlich JR, el Bcheraoui C, el Sayed Zaki M, el-Khatib Z, Elkout H, Elyazar IRF, Enayati A, Endries AY, Er B, Erskine HE, Eshrati B, Eskandarieh S, Esteghamati A, Esteghamati S, Fakhim H, Fallah Omrani V, Faramarzi M, Fareed M, Farhadi F, Farid TA, Farinha CSE, Farioli A, Faro A, Farvid MS, Farzadfar F, Feigin VL, Fentahun N, Fereshtehnejad SM, Fernandes E, Fernandes JC, Ferrari AJ, Feyissa GT, Filip I, Fischer F, Fitzmaurice C, Foigt NA, Foreman KJ, Fox J, Frank TD, Fukumoto T, Fullman N, Fürst T, Furtado JM, Futran ND, Gall S, Ganji M, Gankpe FG, Garcia-Basteiro AL, Gardner WM, Gebre AK, Gebremedhin AT, Gebremichael TG, Gelano TF, Geleijnse JM, Genova-Maleras R, Geramo YCD, Gething PW, Gezae KE, Ghadiri K, Ghasemi Falavarjani K, Ghasemi-Kasman M, Ghimire M, Ghosh R, Ghoshal AG, Giampaoli S, Gill PS, Gill TK, Ginawi IA, Giussani G, Gnedovskaya EV, Goldberg EM, Goli S, Gómez-Dantés H, Gona PN, Gopalani SV, Gorman TM, Goulart AC, Goulart BNG, Grada A, Grams ME, Grosso G, Gugnani HC, Guo Y, Gupta PC, Gupta R, Gupta R, Gupta T, Gyawali B, Haagsma JA, Hachinski V, Hafezi-Nejad N, Haghparast Bidgoli H, Hagos TB, Hailu GB, Haj-Mirzaian A, Haj-Mirzaian A, Hamadeh RR, Hamidi S, Handal AJ, Hankey GJ, Hao Y, Harb HL, Harikrishnan S, Haro JM, Hasan M, Hassankhani H, Hassen HY, Havmoeller R, Hawley CN, Hay RJ, Hay SI, Hedayatizadeh-Omran A, Heibati B, Hendrie D, Henok A, Herteliu C, Heydarpour S, Hibstu DT, Hoang HT, Hoek HW, Hoffman HJ, Hole MK, Homaie Rad E, Hoogar P, Hosgood HD, Hosseini SM, Hosseinzadeh M, Hostiuc M, Hostiuc S, Hotez PJ, Hoy DG, Hsairi M, Htet AS, Hu G, Huang JJ, Huynh CK, Iburg KM, Ikeda CT, Ileanu B, Ilesanmi OS, Iqbal U, Irvani SSN, Irvine CMS, Islam SMS, Islami F, Jacobsen KH, Jahangiry L, Jahanmehr N, Jain SK, Jakovljevic M, Javanbakht M, Jayatilleke AU, Jeemon P, Jha RP, Jha V, Ji JS, Johnson CO, Jonas JB, Jozwiak JJ, Jungari SB, Jürisson M, Kabir Z, Kadel R, Kahsay A, Kalani R, Kanchan T, Karami M, Karami Matin B, Karch A, Karema C, Karimi N, Karimi SM, Kasaeian A, Kassa DH, Kassa GM, Kassa TD, Kassebaum NJ, Katikireddi SV, Kawakami N, Karyani AK, Keighobadi MM, Keiyoro PN, Kemmer L, Kemp GR, Kengne AP, Keren A, Khader YS, Khafaei B, Khafaie MA, Khajavi A, Khalil IA, Khan EA, Khan MS, Khan MA, Khang YH, Khazaei M, Khoja AT, Khosravi A, Khosravi MH, Kiadaliri AA, Kiirithio DN, Kim CI, Kim D, Kim P, Kim YE, Kim YJ, Kimokoti RW, Kinfu Y, Kisa A, Kissimova-Skarbek K, Kivimäki M, Knudsen AKS, Kocarnik JM, Kochhar S, Kokubo Y, Kolola T, Kopec JA, Kosen S, Kotsakis GA, Koul PA, Koyanagi A, Kravchenko MA, Krishan K, Krohn KJ, Kuate Defo B, Kucuk Bicer B, Kumar GA, Kumar M, Kyu HH, Lad DP, Lad SD, Lafranconi A, Lalloo R, Lallukka T, Lami FH, Lansingh VC, Latifi A, Lau KMM, Lazarus JV, Leasher JL, Ledesma JR, Lee PH, Leigh J, Leung J, Levi M, Lewycka S, Li S, Li Y, Liao Y, Liben ML, Lim LL, Lim SS, Liu S, Lodha R, Looker KJ, Lopez AD, Lorkowski S, Lotufo PA, Low N, Lozano R, Lucas TCD, Lucchesi LR, Lunevicius R, Lyons RA, Ma S, Macarayan ERK, Mackay MT, Madotto F, Magdy Abd el Razek H, Magdy Abd el Razek M, Maghavani DP, Mahotra NB, Mai HT, Majdan M, Majdzadeh R, Majeed A, Malekzadeh R, Malta DC, Mamun AA, Manda AL, Manguerra H, Manhertz T, Mansournia MA, Mantovani LG, Mapoma CC, Maravilla JC, Marcenes W, Marks A, Martins-Melo FR, Martopullo I, März W, Marzan MB, Mashamba-Thompson TP, Massenburg BB, Mathur MR, Matsushita K, Maulik PK, Mazidi M, McAlinden C, McGrath JJ, McKee M, Mehndiratta MM, Mehrotra R, Mehta KM, Mehta V, Mejia-Rodriguez F, Mekonen T, Melese A, Melku M, Meltzer M, Memiah PTN, Memish ZA, Mendoza W, Mengistu DT, Mengistu G, Mensah GA, Mereta ST, Meretoja A, Meretoja TJ, Mestrovic T, Mezerji NMG, Miazgowski B, Miazgowski T, Millear AI, Miller TR, Miltz B, Mini GK, Mirarefin M, Mirrakhimov EM, Misganaw AT, Mitchell PB, Mitiku H, Moazen B, Mohajer B, Mohammad KA, Mohammadifard N, Mohammadnia-Afrouzi M, Mohammed MA, Mohammed S, Mohebi F, Moitra M, Mokdad AH, Molokhia M, Monasta L, Moodley Y, Moosazadeh M, Moradi G, Moradi-Lakeh M, Moradinazar M, Moraga P, Morawska L, Moreno Velásquez I, Morgado-da-Costa J, Morrison SD, Moschos MM, Mountjoy-Venning WC, Mousavi SM, Mruts KB, Muche AA, Muchie KF, Mueller UO, Muhammed OS, Mukhopadhyay S, Muller K, Mumford JE, Murhekar M, Musa J, Musa KI, Mustafa G, Nabhan AF, Nagata C, Naghavi M, Naheed A, Nahvijou A, Naik G, Naik N, Najafi F, Naldi L, Nam HS, Nangia V, Nansseu JR, Nascimento BR, Natarajan G, Neamati N, Negoi I, Negoi RI, Neupane S, Newton CRJ, Ngunjiri JW, Nguyen AQ, Nguyen HT, Nguyen HLT, Nguyen HT, Nguyen LH, Nguyen M, Nguyen NB, Nguyen SH, Nichols E, Ningrum DNA, Nixon MR, Nolutshungu N, Nomura S, Norheim OF, Noroozi M, Norrving B, Noubiap JJ, Nouri HR, Nourollahpour Shiadeh M, Nowroozi MR, Nsoesie EO, Nyasulu PS, Odell CM, Ofori-Asenso R, Ogbo FA, Oh IH, Oladimeji O, Olagunju AT, Olagunju TO, Olivares PR, Olsen HE, Olusanya BO, Ong KL, Ong SK, Oren E, Ortiz A, Ota E, Otstavnov SS, Øverland S, Owolabi MO, P A M, Pacella R, Pakpour AH, Pana A, Panda-Jonas S, Parisi A, Park EK, Parry CDH, Patel S, Pati S, Patil ST, Patle A, Patton GC, Paturi VR, Paulson KR, Pearce N, Pereira DM, Perico N, Pesudovs K, Pham HQ, Phillips MR, Pigott DM, Pillay JD, Piradov MA, Pirsaheb M, Pishgar F, Plana-Ripoll O, Plass D, Polinder S, Popova S, Postma MJ, Pourshams A, Poustchi H, Prabhakaran D, Prakash S, Prakash V, Purcell CA, Purwar MB, Qorbani M, Quistberg DA, Radfar A, Rafay A, Rafiei A, Rahim F, Rahimi K, Rahimi-Movaghar A, Rahimi-Movaghar V, Rahman M, Rahman MH, Rahman MA, Rahman SU, Rai RK, Rajati F, Ram U, Ranjan P, Ranta A, Rao PC, Rawaf DL, Rawaf S, Reddy KS, Reiner RC, Reinig N, Reitsma MB, Remuzzi G, Renzaho AMN, Resnikoff S, Rezaei S, Rezai MS, Ribeiro ALP, Roberts NLS, Robinson SR, Roever L, Ronfani L, Roshandel G, Rostami A, Roth GA, Roy A, Rubagotti E, Sachdev PS, Sadat N, Saddik B, Sadeghi E, Saeedi Moghaddam S, Safari H, Safari Y, Safari-Faramani R, Safdarian M, Safi S, Safiri S, Sagar R, Sahebkar A, Sahraian MA, Sajadi HS, Salam N, Salama JS, Salamati P, Saleem K, Saleem Z, Salimi Y, Salomon JA, Salvi SS, Salz I, Samy AM, Sanabria J, Sang Y, Santomauro DF, Santos IS, Santos JV, Santric Milicevic MM, Sao Jose BP, Sardana M, Sarker AR, Sarrafzadegan N, Sartorius B, Sarvi S, Sathian B, Satpathy M, Sawant AR, Sawhney M, Saxena S, Saylan M, Schaeffner E, Schmidt MI, Schneider IJC, Schöttker B, Schwebel DC, Schwendicke F, Scott JG, Sekerija M, Sepanlou SG, Serván-Mori E, Seyedmousavi S, Shabaninejad H, Shafieesabet A, Shahbazi M, Shaheen AA, Shaikh MA, Shams-Beyranvand M, Shamsi M, Shamsizadeh M, Sharafi H, Sharafi K, Sharif M, Sharif-Alhoseini M, Sharma M, Sharma R, She J, Sheikh A, Shi P, Shibuya K, Shigematsu M, Shiri R, Shirkoohi R, Shishani K, Shiue I, Shokraneh F, Shoman H, Shrime MG, Si S, Siabani S, Siddiqi TJ, Sigfusdottir ID, Sigurvinsdottir R, Silva JP, Silveira DGA, Singam NSV, Singh JA, Singh NP, Singh V, Sinha DN, Skiadaresi E, Slepak ELN, Sliwa K, Smith DL, Smith M, Soares Filho AM, Sobaih BH, Sobhani S, Sobngwi E, Soneji SS, Soofi M, Soosaraei M, Sorensen RJD, Soriano JB, Soyiri IN, Sposato LA, Sreeramareddy CT, Srinivasan V, Stanaway JD, Stein DJ, Steiner C, Steiner TJ, Stokes MA, Stovner LJ, Subart ML, Sudaryanto A, Sufiyan M'B, Sunguya BF, Sur PJ, Sutradhar I, Sykes BL, Sylte DO, Tabarés-Seisdedos R, Tadakamadla SK, Tadesse BT, Tandon N, Tassew SG, Tavakkoli M, Taveira N, Taylor HR, Tehrani-Banihashemi A, Tekalign TG, Tekelemedhin SW, Tekle MG, Temesgen H, Temsah MH, Temsah O, Terkawi AS, Teweldemedhin M, Thankappan KR, Thomas N, Tilahun B, To QG, Tonelli M, Topor-Madry R, Topouzis F, Torre AE, Tortajada-Girbés M, Touvier M, Tovani-Palone MR, Towbin JA, Tran BX, Tran KB, Troeger CE, Truelsen TC, Tsilimbaris MK, Tsoi D, Tudor Car L, Tuzcu EM, Ukwaja KN, Ullah I, Undurraga EA, Unutzer J, Updike RL, Usman MS, Uthman OA, Vaduganathan M, Vaezi A, Valdez PR, Varughese S, Vasankari TJ, Venketasubramanian N, Villafaina S, Violante FS, Vladimirov SK, Vlassov V, Vollset SE, Vosoughi K, Vujcic IS, Wagnew FS, Waheed Y, Waller SG, Wang Y, Wang YP, Weiderpass E, Weintraub RG, Weiss DJ, Weldegebreal F, Weldegwergs KG, Werdecker A, West TE, Whiteford HA, Widecka J, Wijeratne T, Wilner LB, Wilson S, Winkler AS, Wiyeh AB, Wiysonge CS, Wolfe CDA, Woolf AD, Wu S, Wu YC, Wyper GMA, Xavier D, Xu G, Yadgir S, Yadollahpour A, Yahyazadeh Jabbari SH, Yamada T, Yan LL, Yano Y, Yaseri M, Yasin YJ, Yeshaneh A, Yimer EM, Yip P, Yisma E, Yonemoto N, Yoon SJ, Yotebieng M, Younis MZ, Yousefifard M, Yu C, Zadnik V, Zaidi Z, Zaman SB, Zamani M, Zare Z, Zeleke AJ, Zenebe ZM, Zhang K, Zhao Z, Zhou M, Zodpey S, Zucker I, Vos T, Murray CJL (2018). Global, regional, and national incidence, prevalence, and years lived with disability for 354 diseases and injuries for 195 countries and territories, 1990–2017: a systematic analysis for the global burden of disease study 2017. Lancet..

[2] Wang Y-P, Chiavegatto AD, Campanha AM, Malik AM, Mogadouro MA, Cambraia M (2017). Patterns and predictors of health service use among people with mental disorders in São Paulo metropolitan area, Brazil. Epidemiol Psychiatr Sci.

[3] Fatori D, Salum GA, Rohde LA, Pan PM, Bressan R, Evans-Lacko S, Polanczyk G, Euripedes M, Graeff-Martins AS. Use of Mental Health Services by Children With Mental Disorders in Two Major Cities in Brazil. Psychiatr Serv. 2019;70(4):337–41. 10.1176/appi.ps.201800389. 10.1176/appi.ps.201800389 30651056

[4] Belmaker RH, Agam G (2008). Major depressive disorder. N Engl J Med.

[5] Kessler RC, Berglund P, Demler O, Jin R, Koretz D, Merikangas KR, Rush AJ, Walters EE, Wang PS, National Comorbidity Survey Replication (2003). The epidemiology of major depressive disorder: results from the National Comorbidity Survey Replication (NCS-R). JAMA..

[6] Gavin NI, Gaynes BN, Lohr KN, Meltzer-Brody S, Gartlehner G, Swinson T (2005). Perinatal depression: a systematic review of prevalence and incidence. Obstet Gynecol.

[7] Gelaye B, Rondon MB, Araya R, Williams MA (2016). Epidemiology of maternal depression, risk factors, and child outcomes in low-income and middle-income countries. Lancet Psychiatry.

[8] Bauer A, Parsonage M, Knapp M, Iemmi V, Adelaja B. The costs of perinatal mental health problems. London School of Economics; 2014. http://everyonesbusiness.org.uk/wp-content/uploads/2014/12/Embargoed-20th-Oct-Summary-of-Economic-Report-costs-of-Perinatal-Mental-Health-problems.pdf. Accessed 21 Sept 2020

[9] Gonçalves H, Pearson RM, Horta BL, González-Chica DA, Castilho E, Damiani M, Lima RC, Gigante DP, Barros FC, Stein A, Victora CG (2016). Maternal depression and anxiety predicts the pattern of offspring symptoms during their transition to adulthood. Psychol Med.

[10] Grote NK, Bridge JA, Gavin AR, Melville JL, Iyengar S, Katon WJ (2010). A meta-analysis of depression during pregnancy and the risk of preterm birth, low birth weight, and intrauterine growth restriction. Arch Gen Psychiatry.

[11] Kingston D, Tough S, Whitfield H (2012). Prenatal and postpartum maternal psychological distress and infant development: a systematic review. Child Psychiatry Hum Dev.

[12] Matijasevich A, Murray J, Cooper PJ, Anselmi L, Barros AJD, Barros FC, Santos IS (2015). Trajectories of maternal depression and offspring psychopathology at 6 years: 2004 Pelotas cohort study. J Affect Disord.

[13] Netsi E, Pearson RM, Murray L, Cooper P, Craske MG, Stein A (2018). Association of persistent and severe postnatal depression with child outcomes. JAMA Psychiatry..

[14] Surkan PJ, Ettinger AK, Ahmed S, Minkovitz CS, Strobino D (2012). Impact of maternal depressive symptoms on growth of preschool- and school-aged children. Pediatrics..

[15] Surkan PJ, Kennedy CE, Hurley KM, Black MM (2011). Maternal depression and early childhood growth in developing countries: systematic review and meta-analysis. Bull World Health Organ.

[16] Alwan S, Friedman JM (2009). Safety of selective serotonin reuptake inhibitors in pregnancy. CNS Drugs.

[17] Lugo-Candelas C, Cha J, Hong S, Bastidas V, Weissman M, Fifer WP, Myers M, Talati A, Bansal R, Peterson BS, Monk C, Gingrich JA, Posner J (2018). Associations between brain structure and connectivity in infants and exposure to selective serotonin reuptake inhibitors during pregnancy. JAMA Pediatr.

[18] Reefhuis J, Devine O, Friedman JM, Louik C, Honein MA, National Birth Defects Prevention Study (2015). Specific SSRIs and birth defects: Bayesian analysis to interpret new data in the context of previous reports. BMJ.

[19] Ammerman RT, Putnam FW, Altaye M, Stevens J, Teeters AR, Van Ginkel JB (2013). A clinical trial of in-home CBT for depressed mothers in home visitation. Behav Ther.

[20] Milgrom J, Negri LM, Gemmill AW, McNeil M, Martin PR (2005). A randomized controlled trial of psychological interventions for postnatal depression. Br J Clin Psychol.

[21] Silverstein M, Diaz-Linhart Y, Cabral H, Beardslee W, Hegel M, Haile W, Sander J, Patts G, Feinberg E (2017). Efficacy of a maternal depression prevention strategy in head start: a randomized clinical trial. JAMA Psychiatry.

[22] Spinelli MG, Endicott J (2003). Controlled clinical trial of interpersonal psychotherapy versus parenting education program for depressed pregnant women. Am J Psychiatry.

[23] Pan American Health Organization. Mental Health Atlas of The Americas. Pan American Health Organization; 2015. http://iris.paho.org/xmlui/bitstream/handle/123456789/28451/9789275119006_eng.pdf?sequence=1&isAllowed=y. Accessed 21 Sept 2020

[24] World Health Organization. WHO | Mental Health Atlas 2014. WHO. 2015. http://www.who.int/mental_health/evidence/atlas/mental_health_atlas_2014/en/. Accessed 11 Sep 2016.

[25] Kazdin AE (2019). Annual research review: expanding mental health services through novel models of intervention delivery. J Child Psychol Psychiatry.

[26] Statista. Number of smartphone users worldwide 2014–2020 | Statista. Statista. 2016. https://www.statista.com/statistics/330695/number-of-smartphone-users-worldwide/. Accessed 15 May 2018.

[27] Statista. Smartphone average selling price worldwide 2010–2019 | Statistic. Statista. 2015. https://www.statista.com/statistics/484583/global-average-selling-price-smartphones/. Accessed 16 May 2018.

[28] Firth J, Torous J, Nicholas J, Carney R, Pratap A, Rosenbaum S, Sarris J (2017). The efficacy of smartphone-based mental health interventions for depressive symptoms: a meta-analysis of randomized controlled trials. World Psychiatry.

[29] Huguet A, Rao S, McGrath PJ, Wozney L, Wheaton M, Conrod J (2016). A systematic review of cognitive behavioral therapy and behavioral activation apps for depression. PLoS One.

[30] Domhardt M, Geßlein H, von Rezori RE, Baumeister H (2018). Internet- and mobile-based interventions for anxiety disorders: a meta-analytic review of intervention components. Depress Anxiety.

[31] Espie CA, Emsley R, Kyle SD, Gordon C, Drake CL, Siriwardena AN, Cape J, Ong JC, Sheaves B, Foster R, Freeman D, Costa-Font J, Marsden A, Luik AI (2018). Effect of digital cognitive behavioral therapy for insomnia on health, psychological well-being, and sleep-related quality of life: a randomized clinical trial. JAMA Psychiatry..

[32] Bostock S, Crosswell AD, Prather AA, Steptoe A (2019). Mindfulness on-the-go: effects of a mindfulness meditation app on work stress and well-being. J Occup Health Psychol.

[33] Chan KL, Chen M (2019). Effects of social media and mobile health apps on pregnancy care: meta-analysis. JMIR mHealth and uHealth.

[34] Cheng H-Y, Huang T-Y, Chien L-Y, Cheng Y-F, Chen F-J (2016). The effects of a mobile application social support program on postpartum perceived stress and depression. Hu Li Za Zhi.

[35] Chan KL, Leung WC, Tiwari A, Or KL, Ip P (2019). Using smartphone-based psychoeducation to reduce postnatal depression among first-time mothers: randomized controlled trial. JMIR Mhealth Uhealth.

[36] Kwong W (2015). What is government’s role in medical apps?. CMAJ..

[37] Bates DW, Landman A, Levine DM (2018). Health apps and health policy: what is needed?. JAMA..

[38] PHP Manual: Rand function. PHP. https://www.php.net/manual/en/function.rand.php. Accessed 10 Jul 2020.

[39] Santos IS, Matijasevich A, Tavares BF, Barros AJD, Botelho IP, Lapolli C, Magalhães PVS, Barbosa APPN, Barros FC (2007). Validation of the Edinburgh Postnatal Depression Scale (EPDS) in a sample of mothers from the 2004 Pelotas Birth Cohort Study. Cad Saude Publica.

[40] Santos IS, Tavares BF, Munhoz TN, Manzolli P, de Ávila GB, Jannke E, Matijasevich A (2016). Patient health questionnaire-9 versus Edinburgh postnatal depression scale in screening for major depressive episodes: a cross-sectional population-based study. BMC Res Notes.

[41] Matijasevich A, Munhoz TN, Tavares BF, Barbosa APPN, da Silva DM, Abitante MS, Dall’Agnol TA, Santos IS (2014). Validation of the Edinburgh Postnatal Depression Scale (EPDS) for screening of major depressive episode among adults from the general population. BMC Psychiatry.

[42] Plummer F, Manea L, Trepel D, McMillan D (2016). Screening for anxiety disorders with the GAD-7 and GAD-2: a systematic review and diagnostic metaanalysis. Gen Hosp Psychiatry.

[43] Moreno AL, DeSousa DA, Souza AMFLP, Manfro GG, Salum GA, Koller SH (2016). Factor structure, reliability, and item parameters of the Brazilian-Portuguese version of the GAD-7 Questionnaire. Temas Psicol.

[44] Henrique IFS, De Micheli D, de Lacerda RB, de Lacerda LA, Formigoni MLO de S. (2004). Validação da versão brasileira do teste de triagem do envolvimento com álcool, cigarro e outras substâncias (ASSIST). Rev Assoc Med Bras.

[45] Siqueira Reis R, Ferreira Hino AA, Romélio Rodriguez Añez C (2010). Perceived Stress Scale. J Health Psychol.

[46] Woods SA, Hampson SE (2005). Measuring the big five with single items using a bipolar response scale. Eur J Personal.

[47] Snyder E, Cai B, DeMuro C, Morrison MF, Ball W (2018). A new single-item sleep quality scale: results of psychometric evaluation in patients with chronic primary insomnia and depression. J Clin Sleep Med.

[48] Craig CL, Marshall AL, Sjöström M, Bauman AE, Booth ML, Ainsworth BE (2003). International physical activity questionnaire: 12-country reliability and validity. Med Sci Sports Exerc.

[49] Lear SA, Hu W, Rangarajan S, Gasevic D, Leong D, Iqbal R, Casanova A, Swaminathan S, Anjana RM, Kumar R, Rosengren A, Wei L, Yang W, Chuangshi W, Huaxing L, Nair S, Diaz R, Swidon H, Gupta R, Mohammadifard N, Lopez-Jaramillo P, Oguz A, Zatonska K, Seron P, Avezum A, Poirier P, Teo K, Yusuf S (2017). The effect of physical activity on mortality and cardiovascular disease in 130 000 people from 17 high-income, middle-income, and low-income countries: the PURE study. Lancet..

[50] Abbott RA, Ploubidis GB, Huppert FA, Kuh D, Croudace TJ (2010). An evaluation of the precision of measurement of Ryff’s psychological well-being scales in a population sample. Soc Indic Res.

[51] Ryff CD, Keyes CL (1995). The structure of psychological well-being revisited. J Pers Soc Psychol.

[52] Ryff CD, Singer BH (2008). Know thyself and become what you are: a eudaimonic approach to psychological well-being. J Happiness Stud.

[53] de Lara MW, Ruschel Bandeira D, Pawlowski J (2013). Validação da Psychological Well-being Scale em uma amostra de estudantes universitários. Avaliação Psicológica.

[54] Damásio BF, Andrade TF, Koller SH (2015). Psychometric properties of the Brazilian 12-item short-form health survey version 2 (SF-12v2). Paidéia (Ribeirão Preto).

[55] Kortum P, Sorber M (2015). Measuring the usability of Mobile applications for phones and tablets. Int J Hum Comput Interact.

[56] Stoyanov SR, Hides L, Kavanagh DJ, Zelenko O, Tjondronegoro D, Mani M (2015). Mobile app rating scale: a new tool for assessing the quality of health mobile apps. JMIR Mhealth Uhealth..

[57] Moreira RS, Magalhães LDC, Siqueira CM, Alves CRL (2019). Adaptação Transcultural do instrumento de vigilância do desenvolvimento infantil “Survey of Wellbeing of Young Children (SWYC)” no contexto brasileiro. J Hum Growth Dev.

[58] Arroll B, Goodyear-Smith F, Crengle S, Gunn J, Kerse N, Fishman T, Falloon K, Hatcher S (2010). Validation of PHQ-2 and PHQ-9 to screen for major depression in the primary care population. Ann Fam Med.

[59] Munhoz TN, Nunes BP, Wehrmeister FC, Santos IS, Matijasevich A (2016). A nationwide population-based study of depression in Brazil. J Affect Disord.

[60] Pallesen S, Bjorvatn B, Nordhus IH, Sivertsen B, Hjørnevik M, Morin CM (2008). A new scale for measuring insomnia: the Bergen insomnia scale. Perceptual Motor Skilk.

[61] American Psychiatric Association (2000). Diagnostic And Statistical Manual Of Mental Disorders DSM-IV-TR Fourth Edition (Text Revision).

[62] da Silva FG (2013). Reconhecimento de movimentos humanos utilizando um acelerômetro e inteligência computacional. Master.

[63] Kirwan M, Duncan MJ, Vandelanotte C, Mummery WK (2012). Using smartphone technology to monitor physical activity in the 10,000 steps program: a matched case-control trial. J Med Internet Res.

[64] Tudor-Locke C, Craig CL, Brown WJ, Clemes SA, De Cocker K, Giles-Corti B (2011). How many steps/day are enough? For adults. Int J Behav Nutr Phys Act.

[65] Edwards EA, Lumsden J, Rivas C, Steed L, Edwards LA, Thiyagarajan A, Sohanpal R, Caton H, Griffiths CJ, Munafò MR, Taylor S, Walton RT (2016). Gamification for health promotion: systematic review of behaviour change techniques in smartphone apps. BMJ Open.

[66] Sardi L, Idri A, Fernández-Alemán JL (2017). A systematic review of gamification in e-health. J Biomed Inform.

[67] Ferster CB (1973). A functional anlysis of depression. Am Psychol.

[68] Lewinsohn PM, Hoberman H, Teri L, Hautzinger M, Reiss S, Bootzin RR (1985). An integrative theory of depression. Theoretical Issues in Behavior Therapy.

[69] Dimidjian S, Barrera M, Martell C, Muñoz RF, Lewinsohn PM (2011). The origins and current status of behavioral activation treatments for depression. Annu Rev Clin Psychol.

[70] Mazzucchelli T, Kane R, Rees C (2009). Behavioral activation treatments for depression in adults: a meta-analysis and review. Clin Psychol Sci Pract.

[71] Ekers D, Webster L, Van Straten A, Cuijpers P, Richards D, Gilbody S (2014). Behavioural activation for depression; an update of meta-analysis of effectiveness and sub group analysis. PLoS One.

[72] Kanter JW, Manos RC, Bowe WM, Baruch DE, Busch AM, Rusch LC (2010). What is behavioral activation?: a review of the empirical literature. Clin Psychol Rev.

[73] Cuijpers P, Andersson G, Donker T, van Straten A (2011). Psychological treatment of depression: results of a series of meta-analyses. Nord J Psychiatry.

[74] Cuijpers P, van Straten A, Andersson G, van Oppen P (2008). Psychotherapy for depression in adults: a meta-analysis of comparative outcome studies. J Consult Clin Psychol.

[75] Dimidjian S, Goodman SH, Sherwood NE, Simon GE, Ludman E, Gallop R, Welch SS, Boggs JM, Metcalf CA, Hubley S, Powers JD, Beck A (2017). A pragmatic randomized clinical trial of behavioral activation for depressed pregnant women. J Consult Clin Psychol.

[76] Moradveisi L, Huibers MJH, Renner F, Arasteh M, Arntz A (2013). Behavioural activation v. antidepressant medication for treating depression in Iran: randomised trial. Br J Psychiatry.

[77] Dobson KS, Hollon SD, Dimidjian S, Schmaling KB, Kohlenberg RJ, Gallop RJ, Rizvi SL, Gollan JK, Dunner DL, Jacobson NS (2008). Randomized trial of behavioral activation, cognitive therapy, and antidepressant medication in the prevention of relapse and recurrence in major depression. J Consult Clin Psychol.

[78] Richards DA, Ekers D, McMillan D, Taylor RS, Byford S, Warren FC, Barrett B, Farrand PA, Gilbody S, Kuyken W, O'Mahen H, Watkins ER, Wright KA, Hollon SD, Reed N, Rhodes S, Fletcher E, Finning K (2016). Cost and outcome of behavioural activation versus cognitive behavioural therapy for depression (COBRA): a randomised, controlled, non-inferiority trial. Lancet..

[79] Dimidjian S, Hollon SD, Dobson KS, Schmaling KB, Kohlenberg RJ, Addis ME, Gallop R, McGlinchey JB, Markley DK, Gollan JK, Atkins DC, Dunner DL, Jacobson NS (2006). Randomized trial of behavioral activation, cognitive therapy, and antidepressant medication in the acute treatment of adults with major depression. J Consult Clin Psychol.

[80] Ekers D, Godfrey C, Gilbody S, Parrott S, Richards DA, Hammond D, Hayes A (2011). Cost utility of behavioural activation delivered by the non-specialist. Br J Psychiatry.

[81] Ekers D, Richards D, McMillan D, Bland JM, Gilbody S (2011). Behavioural activation delivered by the non-specialist: phase II randomised controlled trial. Br J Psychiatry.

[82] Dahne J, Kustanowitz J, Lejuez CW (2018). Development and preliminary feasibility study of a brief behavioral activation mobile application (Behavioral Apptivation) to be used in conjunction with ongoing therapy. Cogn Behav Pract.

[83] Dahne J, Lejuez CW, Kustanowitz J, Felton JW, Diaz VA, Player MS, Carpenter MJ (2017). Moodivate: a self-help behavioral activation mobile app for utilization in primary care-development and clinical considerations. Int J Psychiatry Med.

[84] Martell CR, Dimidjian S, Herman-Dunn R (2013). Behavioral activation for depression: a clinician’s guide.

[85] Lejuez CW, Hopko DR, Acierno R, Daughters SB, Pagoto SL (2011). Ten year revision of the brief behavioral activation treatment for depression: revised treatment manual. Behav Modif.

[86] Perlis ML, Aloia M, Kuhn B (2010). Behavioral treatments for sleep disorders: a comprehensive primer of behavioral sleep medicine interventions (practical resources for the mental health professional). 1 edition.

[87] Hauri PJ (2004). Sleep Disorders.

[88] Chung K-F, Lee C-T, Yeung W-F, Chan M-S, Chung EW-Y, Lin W-L (2018). Sleep hygiene education as a treatment of insomnia: a systematic review and meta-analysis. Fam Pract.

[89] Benson H, Klipper MZ (1975). The relaxation response. Updated & Expanded ed. edition.

[90] Stremler R, Hodnett E, Lee K, MacMillan S, Mill C, Ongcangco L, Willan A (2006). A behavioral-educational intervention to promote maternal and infant sleep: a pilot randomized, controlled trial. Sleep..

[91] Stremler R, Hodnett E, Kenton L, Lee K, Weiss S, Weston J, Willan A (2013). Effect of behavioural-educational intervention on sleep for primiparous women and their infants in early postpartum: multisite randomised controlled trial. BMJ..

[92] Bernstein B, Borkovec T (1973). Progressive Relaxation Training.

[93] Jacobson E (1929). Progressive relaxation: a physiological & clinical investigation of muscular states & their significance in psychology & medical practice. 2nd Edition - Series: The University of Chicago monographs in medicine. edition.

[94] Bhutta ZA, Das JK, Rizvi A, Gaffey MF, Walker N, Horton S, Webb P, Lartey A, Black RE (2013). Evidence-based interventions for improvement of maternal and child nutrition: what can be done and at what cost?. Lancet..

[95] Barker DJP (2012). Sir Richard Doll Lecture. Developmental origins of chronic disease. Public Health.

[96] Benja M, Lawrie TA, Lumbiganon P, Laopaiboon M. Diet or Exercise, or Both, for Preventing Excessive Weight Gain in Pregnancy. Cochrane Database Syst Rev. 2015;6:1–203. 10.1002/14651858.CD007145.pub3. 10.1002/14651858.CD007145.pub3 PMC9428894 26068707

[97] Goldstein RF, Abell SK, Ranasinha S, Misso M, Boyle JA, Black MH, Li N, Hu G, Corrado F, Rode L, Kim YJ, Haugen M, Song WO, Kim MH, Bogaerts A, Devlieger R, Chung JH, Teede HJ (2017). Association of gestational weight gain with maternal and infant outcomes: a systematic review and meta-analysis. JAMA..

[98] Jacka FN, O’Neil A, Opie R, Itsiopoulos C, Cotton S, Mohebbi M, Castle D, Dash S, Mihalopoulos C, Chatterton ML, Brazionis L, Dean OM, Hodge AM, Berk M (2017). A randomised controlled trial of dietary improvement for adults with major depression (the “SMILES” trial). BMC Med.

[99] Lassale C, Batty GD, Baghdadli A, Jacka F, Sánchez-Villegas A, Kivimäki M, Akbaraly T (2018). Healthy dietary indices and risk of depressive outcomes: a systematic review and meta-analysis of observational studies. Mol Psychiatry.

[100] Opie RS, Itsiopoulos C, Parletta N, Sanchez-Villegas A, Akbaraly TN, Ruusunen A, Jacka FN (2017). Dietary recommendations for the prevention of depression. Nutr Neurosci.

[101] Guia Alimentar para a População Brasileira. Ministério da Saúde; 2014.

[102] Secretaria de Atenção à Saúde D de AB. Atenção ao pré-natal de baixo risco. Cadernos de Atenção Básica. 2012.

[103] Institute of Medicine (US) and National Research Council (US) Committee to Reexamine IOM Pregnancy Weight Guidelines (2010). Weight gain during pregnancy: reexamining the guidelines.

[104] World Health Organization (2010). Global recommendations on physical activity for health.

[105] Vargas-Terrones M, Barakat R, Santacruz B, Fernandez-Buhigas I, Mottola MF (2019). Physical exercise programme during pregnancy decreases perinatal depression risk: a randomised controlled trial. Br J Sports Med.

[106] Guthold R, Stevens GA, Riley LM, Bull FC (2018). Worldwide trends in insufficient physical activity from 2001 to 2016: a pooled analysis of 358 population-based surveys with 1·9 million participants. Lancet Glob Health.

[107] Committee on Obstetric Practice (2015). Physical activity and exercise during pregnancy and the postpartum period.

[108] da Silva SG, Ricardo LI, Evenson KR, Hallal PC (2017). Leisure-time physical activity in pregnancy and maternal-child health: a systematic review and meta-analysis of randomized controlled trials and cohort studies. Sports Med.

[109] Bravata DM, Smith-Spangler C, Sundaram V, Gienger AL, Lin N, Lewis R, Stave CD, Olkin I, Sirard JR (2007). Using pedometers to increase physical activity and improve health: a systematic review. JAMA..

[110] Uchino BN (2006). Social support and health: a review of physiological processes potentially underlying links to disease outcomes. J Behav Med.

[111] Elsenbruch S, Benson S, Rücke M, Rose M, Dudenhausen J, Pincus-Knackstedt MK, Klapp BF, Arck PC (2007). Social support during pregnancy: effects on maternal depressive symptoms, smoking and pregnancy outcome. Hum Reprod.

[112] Pfeiffer PN, Heisler M, Piette JD, Rogers MAM, Valenstein M (2011). Efficacy of peer support interventions for depression: a meta-analysis. Gen Hosp Psychiatry.

[113] Mbuagbaw L, Medley N, Darzi AJ, Richardson M, Habiba Garga K, Ongolo-Zogo P (2015). Health system and community level interventions for improving antenatal care coverage and health outcomes. Cochrane Database Syst Rev.

[114] World Health Organization (2016). WHO recommendations on antenatal care for a positive pregnancy experience.

[115] Sockol LE (2015). A systematic review of the efficacy of cognitive behavioral therapy for treating and preventing perinatal depression. J Affect Disord.

[116] Hayes AF (2013). Introduction to mediation, moderation, and conditional process analysis, first edition: a regression-based approach (methodology in the social sciences). First edition.

[117] Cumming G (2011). Cohen’d. Understanding the new statistics: effect sizes, confidence intervals, and meta-analysis.

[118] Harris PA, Taylor R, Thielke R, Payne J, Gonzalez N, Conde JG (2009). Research electronic data capture (REDCap)—a metadata-driven methodology and workflow process for providing translational research informatics support. J Biomed Inform.

[119] Everly GS, Lating JM (2017). The Johns Hopkins Guide to Psychological First Aid. 1 edition.

[120] Naslund JA, Aschbrenner KA, Araya R, Marsch LA, Unützer J, Patel V, Bartels SJ (2017). Digital technology for treating and preventing mental disorders in low-income and middle-income countries: a narrative review of the literature. Lancet Psychiatry.

[121] Menezes P, Quayle J, Garcia Claro H, da Silva S, Brandt LR, Diez-Canseco F, Miranda JJ, Price LSN, Mohr DC, Araya R (2019). Use of a mobile phone app to treat depression comorbid with hypertension or diabetes: a pilot study in Brazil and Peru. JMIR Ment Health..

[122] Brandt LR, Hidalgo L, Diez-Canseco F, Araya R, Mohr DC, Menezes PR, Miranda JJ (2019). Addressing depression comorbid with diabetes or hypertension in resource-poor settings: a qualitative study about user perception of a nurse-supported smartphone app in Peru. JMIR Ment Health.

[123] Evans GW, Cassells RC (2014). Childhood poverty, cumulative risk exposure, and mental health in emerging adults. Clin Psychol Sci.

[124] Evans GW, Kim P (2007). Childhood poverty and health: cumulative risk exposure and stress dysregulation. Psychol Sci.

[125] Roberts G, Bellinger D, McCormick MC (2007). A cumulative risk factor model for early identification of academic difficulties in premature and low birth weight infants. Matern Child Health J.

[126] Torous J, Lipschitz J, Ng M, Firth J (2020). Dropout rates in clinical trials of smartphone apps for depressive symptoms: a systematic review and meta-analysis. J Affect Disord.

[127] Cooper AA, Conklin LR (2015). Dropout from individual psychotherapy for major depression: a meta-analysis of randomized clinical trials. Clin Psychol Rev.

